# A Phase III open-label, randomized, multicenter study of imprime pgg in combination with cetuximab in patients with kras wild type metastatic colorectal cancer

**DOI:** 10.1186/2051-1426-2-S3-P71

**Published:** 2014-11-06

**Authors:** Jeffrey A Meyerhardt, Michele M Grady, Jamie N Lowe, Michele A Gargano, Richard D Huhn, Ada H Braun

**Affiliations:** 1 Dana-Farber Cancer Institute, Harvard Medical School, Boston, MA, USA; 2 Biothera, Eagan, MN, USA

## Background

Single-agent Cetuximab has been shown to improve objective response rate (ORR), progression-free survival (PFS) and overall survival (OS) in patients (pts) with epidermal growth factor receptor (EGFR) expressing, KRAS wild-type (WT) metastatic colorectal cancer (mCRC) who failed Oxaliplatin- and Irinotecan-based therapy or are intolerant to Irinotecan. The mechanism of action of Cetuximab is thought to rely on competitive blockade of endogenous ligand binding and downstream signaling, internalization and down regulation of EGFR, as well as antibody-dependent cellular cytotoxicity (ADCC) (Erbitux SmPC).

Imprime PGG (Imprime) is a novel immune modulator (complex carbohydrate biologic), which harnesses innate immune cells to enhance killing of antibody-targeted tumor cells. In a Phase II single-arm clinical trial in mCRC, the combination of Imprime with Cetuximab resulted in 24% ORR, 62% disease control rate (DCR), and median time to progression (TTP) of 12 wks (Tamayo ME, Ann Onc 2010), representing approximate 100% increases vs historical control (Cunningham, NEJM 2004). ORR was 45%, DCR 82% and TTP 24 wks in pts with KRAS WT tumors (post hoc analysis). The current trial, sponsored by Biothera and registered with http://ClinicalTrials.gov NCT01309126, EudraCT 2010-023562-51, is to confirm these findings in Phase III.

## Trial design

Eligible pts have measurable disease, an ECOG performance status of 0 or 1 and received prior Oxaliplatin- and Irinotecan-based therapy or are intolerant to Irinotecan. Approximately 795 pts will be randomized 2:1 (stratified by geographic region, prior chemotherapy and site) to receive weekly open-label Imprime plus Cetuximab or Cetuximab alone until disease progression. The primary endpoint of the study is OS; secondary endpoints include PFS, ORR (based on RECIST 1.1), quality of life, safety and pharmacokinetics. Exploratory endpoints include biomarker analyses. The primary analysis will occur when ~709 deaths have occurred. Pt screening and enrollment is underway in the United States and Europe.

Trial Registration: http://ClinicalTrials.gov Identifier NCT01309126.

